# Physical Activity, Sedentary Behavior, and Pancreatic Cancer Risk: A Mendelian Randomization Study

**DOI:** 10.1210/jendso/bvae017

**Published:** 2024-02-29

**Authors:** Manuel Gentiluomo, Suzanne C Dixon-Suen, Riccardo Farinella, Giulia Peduzzi, Federico Canzian, Roger L Milne, Brigid M Lynch, Daniele Campa

**Affiliations:** Unit of Genetics, Department of Biology, University of Pisa, Pisa, Italy 56126; Cancer Epidemiology Division, Cancer Council Victoria, Melbourne, Victoria 3004, Australia; Unit of Genetics, Department of Biology, University of Pisa, Pisa, Italy 56126; Unit of Genetics, Department of Biology, University of Pisa, Pisa, Italy 56126; Genomic Epidemiology Group, German Cancer Research Center (DKFZ), Heidelberg, Germany 69120; Cancer Epidemiology Division, Cancer Council Victoria, Melbourne, Victoria 3004, Australia; Centre for Epidemiology and Biostatistics, Melbourne School of Population and Global Health, The University of Melbourne, Melbourne, Victoria 3010, Australia; Precision Medicine, School of Clinical Sciences at Monash Health, Monash University, Clayton, Victoria, Australia 3168; Cancer Epidemiology Division, Cancer Council Victoria, Melbourne, Victoria 3004, Australia; Centre for Epidemiology and Biostatistics, Melbourne School of Population and Global Health, The University of Melbourne, Melbourne, Victoria 3010, Australia; Physical Activity Laboratory, Baker Heart and Diabetes Institute, Melbourne, Victoria, Australia 3004; Unit of Genetics, Department of Biology, University of Pisa, Pisa, Italy 56126

**Keywords:** pancreatic cancer, physical activity, sedentary, Mendelian randomization

## Abstract

Pancreatic cancer is currently the seventh leading cause of cancer death worldwide. Understanding whether modifiable factors increase or decrease the risk of this disease is central to facilitating primary prevention. Several epidemiological studies have described the benefits of physical activity, and the risks associated with sedentary behavior, in relation to cancer. This study aimed to assess evidence of causal effects of physical activity and sedentary behavior on pancreatic cancer risk. We conducted a two-sample Mendelian randomization study using publicly available data for genetic variants associated with physical activity and sedentary behavior traits and genetic data from the Pancreatic Cancer Cohort Consortium (PanScan), the Pancreatic Cancer Case-Control Consortium (PanC4), and the FinnGen study for a total of 10 018 pancreatic cancer cases and 266 638 controls. We also investigated the role of body mass index (BMI) as a possible mediator between physical activity and sedentary traits and risk of developing pancreatic cancer. We found evidence of a causal association between genetically determined hours spent watching television (hours per day) and increased risk of pancreatic cancer for each hour increment (PanScan-PanC4 odds ratio = 1.52, 95% confidence interval 1.17-1.98, *P* = .002). Additionally, mediation analysis showed that genetically determined television-watching time was strongly associated with BMI, and the estimated proportion of the effect of television-watching time on pancreatic cancer risk mediated by BMI was 54%. This study reports the first Mendelian randomization-based evidence of a causal association between a measure of sedentary behavior (television-watching time) and risk of pancreatic cancer and that this is strongly mediated by BMI.

**Summary:** Pancreatic cancer is a deadly disease that is predicted to become the second leading cause of cancer-related deaths by 2030. Physical activity and sedentary behaviors have been linked to cancer risk and survival. However, there is limited research on their correlation with pancreatic cancer. To investigate this, we used a Mendelian randomization approach to examine the genetic predisposition to physical activity and sedentariness and their relation to pancreatic cancer risk, while excluding external confounders. Our findings revealed a causal link between the time spent watching television and an increased risk of pancreatic cancer. Additionally, we determined that over half of the effect of watching television on pancreatic risk is mediated by the individual's BMI.

Pancreatic cancer is the 11th most common tumor and the fifth leading cause of cancer death in Europe and Northern America, and it is projected to become the second by 2030 [1-3]. Pancreatic ductal adenocarcinoma is the most common type of pancreatic cancer, for which only a small number of environmental and genetics risk factors have been identified, making risk stratification and prevention very challenging [4-9]. A key aspect of primary prevention is determining whether modifiable lifestyle factors contribute to an increased or decreased risk of developing the disease. Epidemiological studies have described the benefits of physical activity in relation to cancer risk [10, 11], and there is some evidence suggesting that sedentary behaviors contribute to an increased risk of cancer [12, 13]. For pancreatic cancer, the results obtained so far are inconclusive despite several studies being performed [11, 14-16].

Pancreatic cancer risk factors such as body mass index (BMI), other measures of adiposity, diabetes, insulin resistance, DNA damage, and chronic inflammation may be influenced by physical activity and sedentary habits [17-25]. The presence of these related factors creates a challenge in studying the relationship between physical activity and sedentary behaviors with the risk of developing pancreatic cancer [26]. In recent years, the Mendelian randomization (MR) approach was developed to assess evidence of a causal relationship between an exposure and an outcome, excluding the effect of external confounders [27]. This is possible because MR simulates randomized controlled trials using genotypes as instrumental variables (IVs) or proxies for the exposure of interest [28]. As genotypes are randomly assigned at meiosis, reverse causation is avoided and confounding is minimized, providing evidence for causal inference [29]. The MR approach, under specific assumptions, has proven to be effective in studying the potential causes of pancreatic cancer, consistently showing the causal relationship between BMI, fasting insulin, and low-density lipoprotein levels with pancreatic cancer risk [30]. It has also provided evidence of causal effects of physical activity on reduced risk of various types of cancer, including breast, lung, and ovarian cancer [31-33].

Based on the premise that physical activity and sedentary behaviors are modifiable and present a significant opportunity to reduce the risk of pancreatic cancer in the population, we performed a MR approach to assess the potential causality of the association between 8 physical activity and sedentary-related traits and the risk of pancreatic cancer.

## Material and Methods

### Study Design

The STrengthening the Reporting of OBservational studies in Epidemiology specific for MR guidelines were used in the design of this study.

**Figure 1.**
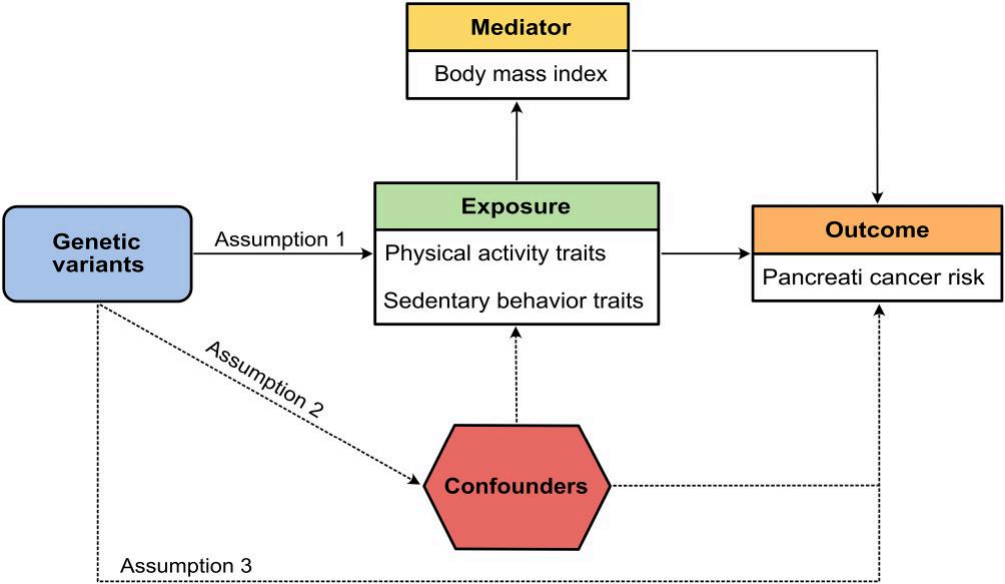
MR study design showing the core assumptions. Principles of MR: assumption 1 (relevance) implies that the genetic variants must have a robust strong correlation with the exposure. Assumption 2 (independence) implies that the genetic variants must be independent of the confounding factors. Assumption 3 (exclusivity) implies that the genetic variants should only influence the outcome through the exposure rather than other means (for example, confounding factors). Dashed lines indicate the deviation from the MR assumption, which should be considered. Abbreviations: MR, Mendelian randomization.

To assess the causal role of physical activity in pancreatic cancer aetiology, a two-sample MR analysis was performed using publicly available data. Specifically, a genome-wide association studies (GWAS) catalog for the genetic instrument data, genotyping data of the PanScan I-III and the PanC4 that are available through the database of Genotypes and Phenotypes (study accession nos. phs000206.v5.p3 and phs000648.v1.p1; project reference no. 12644), and finally summary statistics from the FinnGen study. To respect the 3 core MR assumptions (relevance, independence, and exclusion restriction), the MR design reported in Fig. 1 was strictly followed. Specifically, assumption 1 states that all genetic variants used as instrumental variables should be associated with the exposure of interest; assumption 2 states that none of the IVs should be associated with risk factors and confounders; assumption 3 states that the IVs should affect the outcome only through the exposure, without passing through alternative paths. The potential influence of weak instrument bias was handled by selecting only IVs associated with the related exposure at a genome-wide level of statistical significance (5 × 10^−8^) and by adopting several MR methods undertaking different modeling assumptions such as inverse-variance weighted (IVW) and weighted median (WM).

Three different populations comprising European ancestry individuals without sample overlap were used in this study. Therefore, no significant differences are expected in the association between the genetic variants and the exposure in the exposure and outcome samples.

Additional details on each population, data extraction procedures, and IVs selection are given in the following sections.

### Description of the Exposures and the IVs

The IVs for overall activity, sedentary time, vigorous physical activity, self-reported moderate to vigorous intensity physical activity (MVPA) during leisure time, sedentary behavior at work, time spent watching television, using a computer, and driving were identified using 4 GWAS [34-37]. In the original publications, these 8 traits were evaluated as follows.

Overall activity was assessed with an accelerometer as the average vector magnitude (milligravities per 30-second period), with 1 SD corresponding to ∼50 minutes of moderate activity (eg, brisk walking) per week [36, 38]. An additional set of single-nucleotide polymorphisms (SNPs) identified in a second GWAS on accelerometer-based overall activity was also used, as reported in previous studies [31, 35]. Sedentary time was defined as the ratio of the time spent on sedentary behaviors to the total period for which the accelerometer was worn, considering 30-second intervals [36, 39], and vigorous activity was measured with an accelerometer as the fraction of 30-second intervals containing accelerations over 425 milligravities and using self-reported data (engagement in vigorous activity for at least 10 minutes ≥3 vs 0 days/week) [35, 40]. MVPA was defined as at least 30 minutes per week of MVPA (yes/no) derived from self-reported data in the Mount Sinai BioMe BioBank [37]. Sedentary behavior at work was expressed as self-reported time spent mostly sitting without heavy lifting at work—this value was estimated in the study of Wang and colleagues, aggregating 51 studies with questionnaire-based data [37]. Time spent watching television, using a computer, and driving were all derived from self-reported data within the UK Biobank [34].

Each trait was represented by IVs selected from a unique study. The only exception was the time spent watching television, for which 2 sets of IVs were identified from 2 different studies [36, 37]. To reduce possible bias due to the heterogeneity between the 2 sources of IVs, the largest set was analyzed first, and then a set with all the IVs combined was used.

Adjustment variables included age, sex, and a varying number of principal components depending on each single study. Some studies also adjusted for age-squared, BMI, and genotyping technology or batches.

The list of the 383 selected IVs and the respective sources are reported in Supplementary Table S1 [41].

### Pancreatic Cancer Data

The association between the selected IVs and the risk of developing pancreatic cancer was estimated using the genomic data of 4 pancreatic cancer GWAS: 3 from the PanScan I-III and 1 from the PanC4 [42-45], all available through the database of Genotypes and Phenotypes (study accession nos. phs000206.v5.p3 and phs000648.v1.p1; project reference no. 12644). The 4 datasets were imputed separately using the Michigan Imputation Server and the genotypes of the Haplotype Reference Consortium (HRC r1.1) as reference panel. Before imputation, the datasets were filtered, removing individuals with sex mismatches, call rate <90%, minimal or excessive heterozygosity (>3 SD from the mean), or cryptic relatedness (PI_HAT > 0.2). SNPs with minor allele frequency < 0.005, call rate <90%, or evidence for violations of the Hardy-Weinberg equilibrium (*P* < 1 × 10^−6^) were excluded. After imputation, SNPs with low imputation quality (INFO score r^2^ < 0.7, minor allele frequency < 0.005) were excluded, and then the imputed datasets were merged. A total of 7 543 430 SNPs passed quality controls, and 8769 cases and 7055 controls of European ancestry were used in the analysis. Adjustment covariates included age, sex, and the top 8 principal components.

Of the 383 IVs selected, 305 (80%) were present in the imputed pancreatic cancer case-control dataset and were included in the MR analyses.

Additionally, the summary statistics of a pancreatic cancer GWAS from the FinnGen study that included 1249 pancreatic cancer cases and 259 583 noncancer controls were also used [46]. Adjustment variables included age, sex, and the top 10 principal components.

In total, 325 of the selected IVs were present in the Finnish dataset and included in the MR analyses.

### BMI Data

BMI is strongly associated with physical activity [47] and sedentary behavior. Since it is also a well-known pancreatic cancer risk factor [30], it could be a potential confounder or mediator of the relationship between physical activity and pancreatic cancer risk. Therefore, a mediation analysis was conducted to assess the role of BMI using the summary statistics provided by the Genetic Investigation of ANthropometric Traits (GIANT) consortium [48]. Specifically, GIANT data result from a meta-analysis of 125 studies comprising 322 154 individuals of European ancestry.

### IV Preparation

For each IV, information on reference and alternative alleles and association estimates (betas, standard errors, and *P*-values) were obtained from the original studies that reported the association with the selected traits. The “TwoSampleMR” package was used to clump the IVs at a stringent linkage disequilibrium threshold of r^2^ < 0.01 into a set of independent IVs, using European population genotypes as the reference panel. LDlink was used to identify proxies (r^2^ > 0.99) for the IVs missing from the PanScan-PanC4 imputed datasets or in the FinnGen study [49]. The palindromic IVs (AT or GC) were discarded if the minor allele frequency in the European population was between 45% and 50%. If an IV was reported in the original manuscript with a negative beta, its effect was converted to the positive direction, and, consequently, the effect allele was flipped. The data were harmonized to have the same effect allele for exposure (physical activity/sedentary behavior variables) and outcome (pancreatic cancer risk). Finally, to exclude potential horizontal pleiotropy, IVs were screened for associations with potential confounders (known pancreatic cancer risk factors, arbitrarily selected based on the more recent literature review: personal history of pancreatitis, diabetes, BMI, weight, allergy, lipid levels, dietary habits, and alcohol and tobacco consumption), based on information obtained from GWAS Catalog (data accession February 10, 2023) and PhenoScanner databases [50, 51].

### Statistical Analysis

We performed a two-sample MR analysis using the random effects IVW method to estimate the causal effects of physical activity-related exposures on pancreatic cancer risk [52]. Regression diagnostics based on studentized residuals, hat values, and Cook's distance and leave-one-out analysis were then applied to identify outliers and influential data points, which were considered invalid IVs and were thus removed from the analyses. Then, the possible residual heterogeneity was quantified using Cochran's Q statistic.

To assess the validity of the results and account for the possible presence of undetected invalid IVs, which can affect the results of the IVW analysis, a method with less stringent assumptions like the WM method was performed [53]. The MR-Egger method was carried out to assess directional pleiotropy, and Lasso and MR-PRESSO methods were additionally used for detecting horizontal pleiotropy [54, 55].

Finally, to support the IVW results, the contamination mixture method proposed by Burgess and colleagues was used [56]. This method maintains a low type 1 error rate in the presence of up to 40% nonvalid IVs, conserving a reasonable power to detect a causal effect in all scenarios.

All analyses were repeated, including and excluding the IVs associated with potential confounders identified through the steps described earlier, using the information from GWAS-Catalog and PhenoScanner databases. No adjustment for multiple testing was applied, and the significance threshold was set to *P* = .05. This choice is supported by the guidelines published by Burgess and colleagues, where the authors state that it is not necessary to be overly punitive with multiple testing in MR studies, as they often have moderate power and investigate exposure/outcome relationships that are already supported by epidemiological or biological evidence [57].

**Figure 2.**
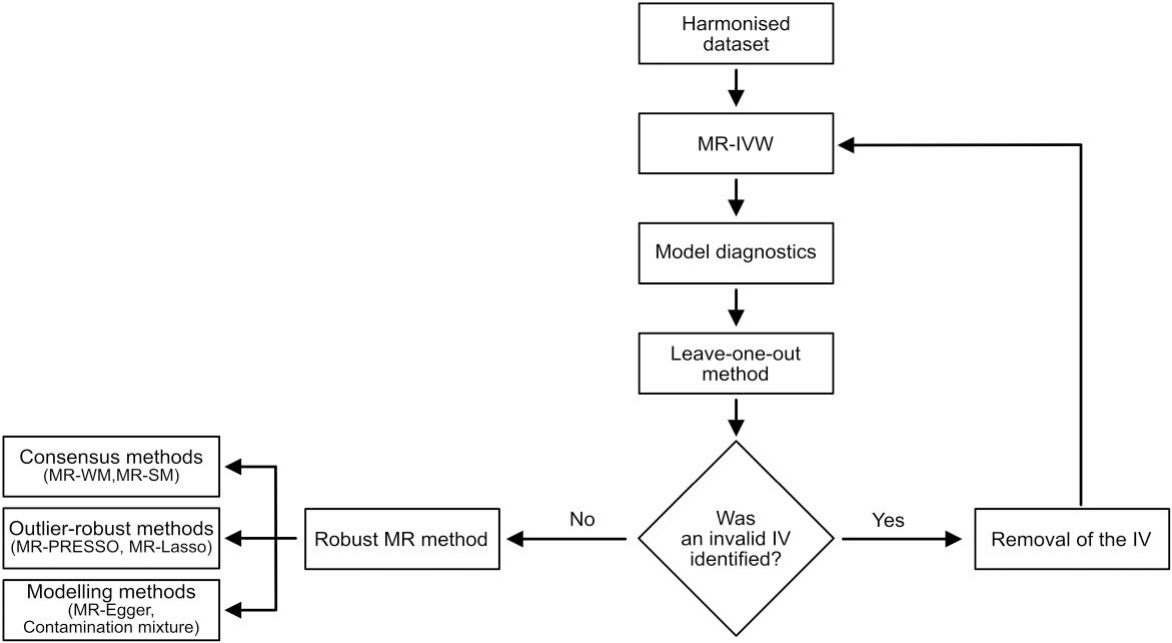
Statistical workflow for the analysis of the causal effects of physical activity and sedentary behavior traits on pancreatic cancer risk. The figure shows the workflow that was adopted for statistical analyses. The IVW method was considered as the reference method for causal estimation. Regression diagnostics, leave-one-out forest plots, and funnel plots were evaluated to identify potential outliers and influential points. Robust methods undertaking different assumptions were also tested to verify the consistency of the IVW results. These models included the simple and weighted median methods, PRESSO, Lasso, Egger, and contamination mixture models. Abbreviations: IVW, inverse-variance weighted.

The workflow for the statistical analysis is reported in Fig. 2. All analyses were two-sided tests and were performed using RStudio version 4.2.2 with the “MendelianRandomization” (0.7.0), “TwoSampleMR” (0.5.6), and “MRPRESSO” (1.0) packages. A two-sided *P* < .05 was considered statistically significant.

### Mediation Analyses

A regression-based multivariable IVW MR approach [58] was used to disentangle further the causal effect of physical activity or sedentary traits and BMI on pancreatic cancer. Specifically, the effect of BMI on pancreatic cancer risk was estimated using the multivariable IVW, adjusting for the effect of the IVs on physical activity or sedentary behavior. Then, the effect of physical activity or sedentary behavior on BMI was estimated using the IVW model. Next, the product method, described in detail elsewhere [59], was applied to estimate the indirect effect of physical activity or sedentary behavior traits on pancreatic cancer risk through BMI, adopting the approach reported by Carter and colleagues [60]. Finally, the proportion of the effect of physical activity or sedentary behavior mediated by BMI was calculated by dividing the indirect effect by the effect of physical activity or sedentary behavior on pancreatic cancer risk.

## Results

The results based on the PanScan-PC4 dataset showed evidence of a causal association between the hours per day spent watching television and an increased risk of developing pancreatic cancer (Table 1). The IVW analysis showed a statistically significant association using 144 valid IVs [odds ratio (OR) = 1.52, 95% confidence interval (CI) = 1.17-1.98, *P* = .002], per each hour spent watching television. The scatter plot, funnel plot, and regression diagnostic plots are reported in Supplementary Figs. S1–S4 [61]. The analysis carried out excluding IVs associated with potential confounders showed the effect, direction, and magnitude in agreement with the results obtained analyzing all the IVs, with an OR = 1.45, 95% CI 1.01-2.10, *P* = .046.

**Table 1.** The causal effect of physical activity and sedentary behavior traits on pancreatic cancer risk

Exposure	n_IV	IVW			Weighted median		Egger		Egger (intercept)		Lasso		PRESSO	
		OR (95% CI)	*P*-value	Cochran's Q *P*-value	OR (95% CI)	*P*-value	OR (95% CI)	*P*-value	estimate	*P*-value	OR (95% CI)	*P*-value	OR (95% CI)	*P*-value
MVPA	5	0.82 (0.35-1.92)	.644	0.324	0.73 (0.24-2.21)	.579	17.35 (0.001-100)	.554	−0.08	.525	0.82 (0.35-1.92)	.644	0.82 (0.40-1.67)	.610
MVPA^a^	3	1.82 (0.74-4.48)	.193	0.591	2.12 (0.75-5.99)	.155	72.29 (0.39-100)	.109	−0.12	.161	1.82 (0.71-4.68)	.214	—	—
OaD	3	0.38 (0.14-1.03)	.058	0.538	0.28 (0.07-1.16)	.079	0 (0-3.50)	.054	1.77	.058	0.38 (0.09-1.53)	.172	—	—
OaD*^a^*	1	1.50 (0.26-8.85)	.653	—	—	—	—	—	—	—	—	—	—	—
OaK	9	0.62 (0.32-1.19)	.151	0.252	0.72 (0.30-1.71)	.459	11.97 (0.77-186.65)	.077	−0.09	**.042**	0.62 (0.32-1.19)	.151	0.62 (0.32-1.19)	.189
OaK*^a^*	4	1.29 (0.51-3.22)	.590	0.872	1.33 (0.46-3.84)	.602	2.60 (0.10-65.45)	.561	−0.03	.655	1.29 (0.51-3.22)	.590	—	—
VpaK	5	0.99 (0.36-2.74)	.983	0.004	1.26 (0.55-2.91)	.586	18.03 (0.01–52 220)	.477	−0.12	.471	0.99 (0.36-2.74)	.983	1.00 (0.40-2.46)	.996
VpaK*^a^*	3	1.00 (0.51-1.95)	.994	0.163	1.22 (0.50-2.99)	.670	13.23 (0.05-3781)	.371	−0.12	.364	1.00 (0.41-2.46)	.996	—	—
SbD	4	0.70 (0.31-1.61)	.405	0.646	0.54 (0.20-1.44)	.214	0.04 (0–9 378 392)	.744	0.08	.771	0.70 (0.31-1.61)	.405	—	—
SbD*^a^*	2	1.01 (0.30-3.37)	.986	0.318	−	—	—	—	—	—	—	—	—	—
Srcu	44	0.66 (0.41-1.08)	.100	0.298	0.59 (0.30-1.13)	.112	0.76 (0.03-18.06)	.866	−0.002	.931	0.66 (0.41-1.08)	.100	0.73 (0.51-1.06)	.105
Srcu*^a^*	27	0.82 (0.44-1.56)	.549	0.482	0.66 (0.28-1.57)	.349	0.97 (0.01-66.36)	.990	−0.003	.937	0.82 (0.44-1.56)	.549	0.82 (0.49-1.37)	.461
Srdt	4	0.45 (0.09-2.21)	.323	0.576	0.36 (0.05-2.39)	.290	0 (0-163.17)	.223	0.12	.268	0.45 (0.09-2.21)	.323	—	—
Srdt*^a^*	2	0.22 (0.02-1.98)	.176	0.643	—	—	—	—	—	—	—	—	—	—
SrSbw	4	0.71 (0.38-1.32)	.281	0.670	0.72 (0.35-1.48)	.367	0.81 (0.09-7.02)	0.847	−.01	0.902	.71 (0.38-1.32)	0.281	—	—
SrSbw*^a^*	2	0.83 (0.27-2.60)	.749	0.416	—	—	—	—	—	—	—	—	—	—
SrTVu_set1	144	1.52 (1.17-1.98)	**.002**	1.00	1.52 (1.05-2.19)	**.025**	2.35 (0.76-7.29)	.138	−0.01	.436	1.52 (1.17-1.98)	**.002**	1.52 (1.26-1.83)	**.00002**
SrTVu_set1*^a^*	86	1.45 (1.01-2.10)	**.046**	1.00	1.42 (0.85-2.34)	.178	2.61 (0.63-10.74)	.184	−0.01	.401	1.45 (1.01-2.10)	**.046**	1.45 (1.11-1.90)	**.009**
SrTVu_set2	177	1.32 (1.07-1.63)	**.010**	0.986	1.33 (0.99-1.80)	.061	1.05 (0.53-2.07)	.901	0.01	.485	1.32 (1.07-1.63)	**.010**	1.32 (1.10-1.59)	**.004**
SrTVu_set2*^a^*	96	1.48 (1.10-1.99)	**.010**	1.00	1.39 (0.91-2.13)	.126	1.22 (0.48-3.07)	0.677	0.004	.660	1.48 (1.10-1.99)	**.010**	1.48 (1.18-1.85)	**.001**

Abbreviations: CI, confidence interval; IV, instrumental variable; IVW, inverse-variance weighted; MVPA, moderate to vigorous physical activity; OaD, overall activity (identified by Doherty et al [35]); OaK, overall activity (identified by Klimentidis et al [32]); OR, odds ratio; PanScan, Pancreatic Cancer Cohort Consortium; PanC4, Pancreatic Cancer Case-Control Consortium; SbD, sedentary behavior (identified by Doherty et al [35]); Srcu, self-reported computer use; Srdt, self-reported driving time; SrSbw, self-reported sedentary behavior at work (identified by Wang et al [34]); SrTVu, self-reported TV use; VpaK, vigorous physical activity.

These results refer to the PanScanI-III + PanC4 dataset for pancreatic cancer risk.

^*a*^Results of the analyses performed excluding the IVs identified as outliers through sensitivity analyses. Results reported in bold are statistically significant (*P* < .05). The causal effect estimated for each physical activity-related trait on pancreatic cancer risk is reported in the form of an OR with its 95% CI, while the estimate for the pleiotropic effect in Egger analysis (Egger's intercept term) is reported without conversion to the OR scale.

Furthermore, “watching television” is the only trait for which the IVs were obtained from 2 different studies. The analysis conducted by combining the IVs of both studies (total of 177 IVs) confirmed the association with increased risk of developing pancreatic cancer as obtained with the other models: OR = 1.32, 95% CI 1.07-1.63, *P* = .010, OR = 1.48, 95% CI 1.10-1.99, *P* = .010.

In addition, the findings obtained from the WM and contamination mixture techniques, particularly the magnitude and direction of the association, agreed with and supported the results of the IVW analyses. The associations for the WM and contamination mixture models were statistically significant when using the complete set of IVs. However, the associations were no longer statistically significant when using the IV set that excludes variants associated with potential confounding factors. Finally, no directional pleiotropy or heterogeneity was detected using the MR-Egger method and Cochran's Q-statistic, respectively (Supplementary Fig. S1 [61] and Table 1), and no outlier IV was identified using the MR-PRESSO method.

The analyses conducted using FinnGen showed an increment of pancreatic cancer risk for each genetically predicted hour spent watching television (OR = 1.84, 95% CI 1.19-2.85, *P* = .006; 169 IVs), albeit with some pleiotropy, as indicated by the MR-Egger analysis (*P* = .027). No statistically significant results were obtained after removing the IVs associated with confounders or when using the extended IVs list obtained by combining the 2 sets of IVs (182 IVs). The results of the analyses conducted for television-watching time are reported in Table 2.

**Table 2.** Causal effect of television-watching time on pancreatic cancer risk

Dataset		PanScanI-III-PC4	FinnGen	PanScanI-III-PC4	FinnGen
Exposure		SrTVu_set1	SrTVu_set1	SrTVu_set2	SrTVu_set2
Number of IVs		144	169	177	182
IVW	OR (95% CI)	1.52 (1.17-1.98)	1.84 (1.19-2.85)	1.32 (1.07-1.63)	1.42 (0.98-2.05)
	*P*-value	**.002**	**.006**	**.010**	.061
IVW*^b^*	OR (95% CI)	1.45 (1.01-2.10)	1.15 (0.63-2.08)	1.48 (1.10-1.99)	0.94 (0.58-1.53)
	*P*-value	**.046**	.649	**.010**	.816
Weighted median	OR (95% CI)	1.52 (1.05-2.19)	2.36 (1.26-4.41)	1.33 (0.99-1.80)	1.56 (0.92-2.64)
	*P*-value	**.025**	**.007**	.061	.096
Weighted median*^b^*	OR (95% CI)	1.42 (0.85-2.34)	1.15 (0.50-2.66)	1.39 (0.91-2.13)	1.24 (0.62-2.47)
	*P*-value	.178	.747	.126	.543
Egger	OR (95% CI)	2.35 (0.76-7.29)	15.61 (2.24-108.73)	1.05 (0.53-2.07)	1.94 (0.56-6.70)
	*P*-value	.138	**.006**	.901	.297
Egger*^b^*	OR (95% CI)	2.61 (0.63-10.74)	1.91 (0.12-30.70)	1.22 (0.48-3.07)	0.66 (0.15-2.94)
	*P*-value	.184	.647	.677	.584
Egger (intercept)	estimate	−0.01	−0.04	0.01	−0.01
	*P*-value	.436	**.027**	.485	.610
Egger (intercept)*^b^*	estimate	−0.01	−0.09	0.004	0.01
	*P*-value	.401	.712	.660	.618
Lasso	OR (95% CI)	1.52 (1.17-1.98)	1.84 (1.19-2.85)	1.32 (1.07-1.63)	1.42 (0.98-2.05)
	*P*-value	**.002**	**.006**	**.010**	.061
Lasso*^b^*	OR (95% CI)	1.45 (1.01-2.10)	1.15 (0.63-2.08)	1.48 (1.10-1.99)	0.94 (0.58-1.53)
	*P*-value	**.046**	.649	**.010**	.816
PRESSO	OR (95% CI)	1.52 (1.26-1.83)	1.84 (1.26-2.70)	1.32 (1.10-1.59)	1.42 (1.03-1.96)
	*P*-value	**.00002**	**.002**	**.004**	**.033**
PRESSO*^b^*	OR (95% CI)	1.45 (1.11-1.90)	1.15 (0.70-1.89)	1.48 (1.18-1.85)	0.94 (0.61-1.46)
	*P*-value	**.009**	.588	**.001**	.798
Contamination mixture	OR (95% CI)	1.67 (1.22-2.61)	2.92 (1.67-5.16)	1.39 (1.07-1.88)	2.44 (1.60-3.78)
	*P*-value	**.005**	**.0031**	**.021**	**.004**
Contamination mixture*^b^*	OR (95% CI)	1.97 (1.40-2.83)	2.53 (1.36-4.53)	1.49 (1.14-2.25)	2.44 (1.60-3.78)
	*P*-value	**.0005**	**.010**	**.004**	**.004**
BMI*^a^*	OR (95% CI)	1.96 (1.10-3.48)	0.79 (0.22-2.84)	1.66 (0.90-3.07)	1.08 (0.41-2.84)
	*P*-value	**.024**	.716	.111	.870

Abbreviations: BMI, body mass index; CI, confidence interval; IV, instrumental variable; IVW, inverse-variance weighted; OR, odds ratio; PanC4, Pancreatic Cancer Case-Control Consortium; PanScan, Pancreatic Cancer Cohort Consortium; SrTVu, self-reported TV use.

All results reported in bold are statistically significant (*P* < .05). The causal effect of self-reported TV watching time on pancreatic cancer risk is reported as OR with its 95% CI.

^*a*^The result is related to the effect of BMI on pancreatic cancer risk in a multivariable Mendelian randomization analysis (IVW method) adjusted for self-reported television-watching time.

^*b*^The analyses were performed excluding the IVs identified as outliers through sensitivity analyses.

The analysis of the other traits did not show any statistically significant association in any of the datasets (Table 1).

**Figure 3.**
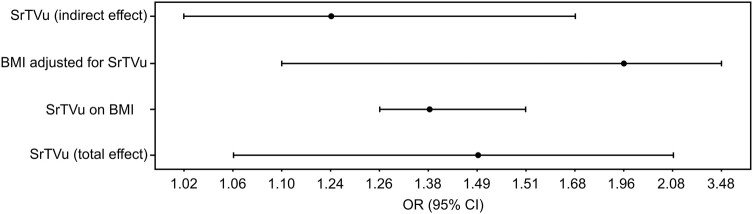
Forest plot for the results of mediation analysis. The figure shows the results of the mediation analysis of BMI in the relationship between television-watching time and pancreatic cancer. The total effect of television-watching time on pancreatic cancer risk (PanScanI-III + PC4 data) was estimated using the IVW approach. The effect of television-watching time on BMI (GIANT data) was estimated using the IVW approach. The effect of BMI (GIANT data) on pancreatic cancer risk (PanScanI-III + PC4 data) adjusted for television-watching time was estimated using a multivariable IVW method. The indirect effect of television-watching time on pancreatic cancer risk through BMI was calculated applying the product method. Abbreviations: BMI, body mass index; GIANT, Genetic Investigation of ANthropometric Traits; IVW, inverse-variance weighted; PanScan, Pancreatic Cancer Cohort Consortium; PanC4, Pancreatic Cancer Case-Control Consortium.

A mediation analysis was conducted to assess the role of BMI in the association between television-watching time and pancreatic cancer risk (using PanScan-PanC4 data); BMI-related summary statistics of the IVs associated with television-watching time were found for a subset of 85 IVs in the GIANT dataset. Using this subset of IVs, the total effect of television-watching time on pancreatic cancer risk (PanScan-PanC4 data) was still statistically significant, with an OR = 1.49, 95% CI 1.06-2.08, *P* = .020. However, in the multivariable IVW MR model, the direct effect of television-watching time on pancreatic cancer risk was not statistically significant (OR = 1.20, 95% CI 0.88-1.63, *P* = .256), whereas the effect of BMI indicated that BMI is associated with an increased risk of pancreatic cancer (OR = 1.96, 95% CI 1.10-3.48, *P* = .022). Furthermore, genetically predicted television-watching time was strongly associated with BMI with an OR = 1.38, 95% CI 1.26-1.51, *P* = 1.92 × 10^−5^, and the indirect effect of genetically predicted television-watching time through BMI was estimated as OR = 1.24, 95% CI = 1.02-1.68 (Fig. 3). The proportion of the effect of genetically predicted television-watching time on pancreatic cancer risk mediated by BMI was estimated to be 54% (34-71%).

## Discussion

Several studies have been published in recent years on the impact of physical activity and sedentary behavior on the risk of developing pancreatic cancer [11, 14-16]. However, these studies did not provide conclusive evidence of the potential benefits of physical activity or the risks associated with a sedentary lifestyle. The MR approach has proven useful in providing insights into and in establishing the causal associations among several exposures and cancer risk [62].

We analysed 8 traits that represent physical activity and sedentary behavior, and the results indicate a role of the time spent watching television in increasing pancreatic cancer risk. The causal effect of television-watching time was supported by the results obtained by applying different models and methods, each characterized by a different level of tolerance to pleiotropy and by the presence of potentially invalid IVs. All models showed concordant results, with identical directions of the effect and similar effect sizes. The association was also supported by the results obtained from FinnGen, which in the more comprehensive model (ie, when all the IVs were included) showed results similar to PanScan. The concordance between the results obtained from 2 different populations supports the generalizability of the results across European populations.

We estimated that just over half (54%) of the effect of television-watching time was mediated by BMI. This result provides additional evidence that BMI is a pancreatic cancer risk factor [30]. The relationship between television-watching time and BMI has been extensively studied in young subjects, albeit with fewer studies in adults. Evidence from prospective observational studies suggests that television viewing is associated with a higher BMI, overweight, obesity, greater waist circumference, and increased adiposity [63-65]. Our findings align with those in the literature supporting a causal relationship between television-viewing time and higher BMI. However, the mechanism that links television-watching time and BMI is not clearly defined. Additionally, apart from the impact of BMI, the duration of time spent watching television can be considered as an indicator of leading a sedentary lifestyle in modern society. This in turn is linked to certain factors that could potentially clarify the link between watching television and the risk of developing pancreatic cancer. In lean individuals, a sedentary behavior is associated with enhanced systemic inflammation and C-reactive protein levels independent from obesity [66]. The presence of systemic inflammatory processes has been reported as potential mediators of pancreatic cancer development and progression [67, 68]. Furthermore, time spent watching television is not only an indicator of a sedentary lifestyle but also of reduced time spent outdoors, and it can lead to less exposure to the sun, which can result in reduced vitamin D production. Vitamin D is primarily obtained through UV irradiation, and those who prefer indoor activities and receive less sun exposure, such as those who watch television, are often observed to have low levels of it [69]. Vitamin D is a well-known regulator of immune system processes that target genes, regulatory transcription factors, and epigenetic modulators to promote anti-inflammatory responses in various types of cancer [70-72]. Additionally, it is suggested as a protective factor against pancreatic cancer [73]. Last, leisure sedentary activities such as watching television are associated with unhealthy eating habits like high intake of heavily burnt meat and sugar-sweetened beverages and low intake of vegetables [74]. Based on a recent review, a healthy plant-based diet with fruits and vegetables is linked to a lower risk of prostate cancer, while a high intake of red meat is associated with a higher risk of developing pancreatic cancer [75].

The lack of association with pancreatic cancer risk for all the other genetically predicted traits is intriguing. However, concerning sedentary behaviors, individuals typically spend more time watching television than using a computer for recreation or driving a car. This makes television-watching time the most representative of these 3 sedentary behaviors [76]. It is also important to consider the number of IVs used for each trait and particularly that only a limited number of IVs were selected for traits other than television-watching time; this reflects the current limited knowledge we have of their genetics. Computer use is the trait with the second largest number of IVs in our study, with 48 available in the PanScan-PanC4 and FinnGen datasets. Based on the type of data used in our study, computer use time is referred exclusively to as recreational use, excluding the sitting and screen time spent at work, as reported in the original study by van de Vegte et al [34] and thus potentially reducing the degree to which this trait represents overall sitting and screen-watching time. Computer use time is often combined with television-viewing time as a single indicator of screen-watching time. However, in some studies, including ours, these traits were analyzed separately, reporting a statistically significant association with television-watching time but not with recreational computer use. For example, van de Vegte and colleagues performed a MR study to evaluate the causal effect on coronary artery disease of several sedentary traits; they reported evidence of a causal relationship for television-viewing time but not for computer use time [34]. An observational study on self-reported recreational screen time (television viewing and recreational computer use) and site-specific cancer risk showed that daily television-viewing time was associated with higher risks of oropharyngeal, esophagogastric, and colon cancer. However, there was no association with computer use [77]. In the same study, consistent with our results, an association with pancreatic cancer risk was observed for self-reported daily television-viewing time, but not self-reported daily computer time, in a model considering only age and sex as adjustment variables.

We observed an association between a genetic predisposition to spend time watching television and an increased susceptibility to pancreatic cancer, with more than half of the effect being attributable to BMI as a mediator of the association. Furthermore, BMI is influenced by the time spent watching television. As reported by Chighaf and colleagues, adolescents with 4 hours or more hours per day (vs <1 hour) of television viewing per day are more likely (OR = 2.19, CI 95% 1.73, 2.77) to be overweight/obese than those who use computers or handheld device in adolescents who spent ≥4 hours (vs <1 hour) on these devices (OR = 1.53, 95% CI 1.19, 1.97) [78].

Based on these findings, the long-term reduction of television-viewing time alone may not be enough to lower the risk of pancreatic cancer per se. The reduction of television-viewing time induces, in part, the reduction of pancreatic cancer risk through the reduction of BMI. In fact, our study suggests that an intervention to reduce television-viewing time would have at least 2 effects: 1 to reduce BMI and another to reduce the risk of pancreatic cancer (some of the latter due to the former).

In conclusion, this first MR analysis of physical activity and sedentary behaviors in relation to pancreatic cancer risk found evidence that a measure of sedentary behavior (greater television-watching time) is causally related to an increased risk of pancreatic cancer and, further, that this is partly due to its causal effect to increase BMI.

## Data Availability

All data included in this study are publicly available in a controlled access repository. The strictly specific summary statistics used to perform the Mendelian randomization analyses in this study are reported in Supplementary Table S1 [41].
